# Longitudinal prediction of falls and near falls frequencies in Parkinson’s disease: a prospective cohort study

**DOI:** 10.1007/s00415-020-10234-6

**Published:** 2020-09-24

**Authors:** Beata Lindholm, Christina Brogårdh, Per Odin, Peter Hagell

**Affiliations:** 1 Department of Clinical Sciences, Clinical Memory Research Unit, Lund University, Malmö, Sweden; 2 Department of Neurology, Rehabilitation Medicine, Memory Disorders, and Geriatrics, Skåne University Hospital, 205 02 Malmö, Sweden; 3 Department of Health Sciences, Lund University, Lund, Sweden; 4 Division of Neurology, Department of Clinical Sciences Lund, Restorative Parkinson Unit, Lund University, Lund, Sweden; 5 The PRO-CARE Group, Faculty of Health Sciences, Kristianstad University, Kristianstad, Sweden

**Keywords:** Prediction, Falls/near falls, Tandem gait, Cognition, Parkinson’s disease

## Abstract

**Introduction and objective:**

Several prediction models for falls/near falls in Parkinson’s disease (PD) have been proposed. However, longitudinal predictors of frequency of falls/near falls are poorly investigated. Therefore, we aimed to identify short- and long-term predictors of the number of falls/near falls in PD.

**Methods:**

A prospective cohort of 58 persons with PD was assessed at baseline (mean age and PD duration, 65 and 3.2 years, respectively) and 3.5 years later. Potential predictors were history of falls and near falls, comfortable gait speed, freezing of gate, dyskinesia, retropulsion, tandem gait (TG), pain, and cognition (Mini-Mental State Exam, MMSE). After each assessment, the participants registered a number of falls/near falls during the following 6 months. Multivariate Poisson regression was used to identify short- and long-term predictors of a number of falls/near falls.

**Results:**

Baseline median (q1–q3) motor (UPDRS) and MMSE scores were 10 (6.75–14) and 28.5 (27–29), respectively. History of falls was the only significant short-time predictor [incidence rate ratio (IRR), 15.17] for the number of falls/near falls during 6 months following baseline. Abnormal TG (IRR, 3.77) and lower MMSE scores (IRR, 1.17) were short-term predictors 3.5 years later. Abnormal TG (IRR, 7.79) and lower MMSE scores (IRR, 1.49) at baseline were long-term predictors of the number of falls/near falls 3.5 years later.

**Conclusion:**

Abnormal TG and MMSE scores predict the number of falls/near falls in short and long term, and may be indicative of disease progression. Our observations provide important additions to the evidence base for clinical fall prediction in PD.

## Introduction

Falls and balance problems in Parkinson disease (PD) are common already in the early stages of disease [1, 2] and progress over time [3]. In unselected populations, an average of 60.5% (35–90%) of persons with PD (PwPD) fell at least once over 12 months, with two-thirds being recurrent fallers [4]. Prospective studies have proposed numerous falls prediction models in PD [5–10]. The most robust predictor for future falls is a history of falls, whereas some observations suggest that a history of near falls has greater clinical value in terms of working proactively to avoid the occurrence of falls [6]. It has also been recommended that prediction models should avoid time-consuming clinical tests and assessments, to facilitate their use in clinical practice [5]. Moreover, a growing body of research highlights the importance of assessing cognition for prediction of falling in PD [11]. The Mini-Mental State Examination (MMSE) is the most commonly used screening tool for global cognitive impairment in clinical settings [12] and lower MMSE scores have been suggested as a predictor of falls in PD [13]. However, the significance of predictors based on a single assessment is uncertain given the impact of disease progression on falling. Therefore, prospective cohort studies are needed [14].

Furthermore, the previously proposed prediction models were derived to discriminate future fallers from non-fallers [5–10] and only a few studies investigated the impact of predictors on the frequency of falling [1, 14, 15]. To the best of our knowledge, no study has assessed predictors of the frequency of near falls. Prediction of the frequency of falls/near falls is of great clinical relevance, since a higher frequency may contribute to increased risk for fractures [16, 17]. Therefore, we aimed to identify short- and long-term predictors of the number of falls and/or near falls in PD.

## Methods

### Ethics statement

The Regional Ethical Review Board in Lund, Sweden approved the study (Dnr 2011/768). All participants gave written informed consent to participate.

### Study design

This prospective cohort study includes two clinical assessments: the baseline assessment (A1) at the start of the study and a second assessment (A2) 3.5 years later. After each assessment, participants were instructed to register all consecutive falls and near falls during the following 6 months; first follow-up (F1) and second follow-up (F2), respectively.

### Participants

All people with PD [18] diagnosed by a movement disorder specialist who received care at a south Swedish university hospital at the start of the study were considered eligible for inclusion in the study (*n* = 349). Exclusion criteria at the start of the study were: age above 80 years (*n* = 116), inability to stand without support, i.e., spontaneous tendency to fall in standing position (*n* = 22), unable to understand instructions (*n* = 14), or having severe comorbidity (*n* = 11). Of the remaining 186 potential participants, 40 (16 women) declined participation. The study sample at A1 included 146 PwPD. At the time for A2, 24 PwPD had dropped out due to severe comorbidity or death and 49 (17 women) declined participation. Severe comorbidity included metastatic cancer*,* stroke, heart failure, dialysis-treated renal failure, age-related eye diseases, gastrointestinal diseases, and diabetic complications. The final study sample with available A1 and A2 data included 73 PwPD.

### Assessment and procedure

Data on demographics and anti-parkinsonian medications were obtained from medical records. Daily levodopa equivalent (LDE) doses (mg/day) were calculated according to recommended conversion factors [19]. At both A1 and A2, all participants were assessed during outpatient visits, which were scheduled at a time of day when the participant usually reported feeling at his/her best. All PwPD self-rated their motor status at the time of examination as “good/on”, “on with dyskinesia”, or “bad/off”. This was followed by a clinical investigation of nine variables (Table 1) constituting potential predictors for future falls/near falls. In addition, motor symptoms were assessed using the Unified PD Rating Scale (UPDRS) part III (score range, 0–108) [21], and PD severity was classified according to Hoehn & Yahr (HY) staging (range, I–V) [25]. All assessments were conducted by the same physical therapist (BL).

**Table 1:** Overview of potential predictors for number of future falls and/or near falls

	Description; score range	Dichotomized	References
Cognition (MMSE)	8-item cognitive screening test; score range 0–30 (higher = better)	Not applicable	[20]
Comfortable gait speed m/s (10MWT)	Time (s) to walk 10 m (without acceleration/deceleration distance) in comfortable gait speed	Normal (≥ 1.1 m/s), low (< 1.1 m/s)	[5, 7, 9, 10]
Dyskinesia (item 32, UPDRS)	Proportion of the waking day with dyskinesias; 0 (none)—4 (76–100%)	No dyskinesias (0), dyskinesias (1–4)	[21]
Freezing of gait (item 3, FOGQsa)	Do you feel that your feet get glued to the floor while walking, making a turn or when trying to initiate walking (freezing)?; 0 (never)—4 (always—whenever walking)	No FOG (0), FOG (1–4)	[22]
History of falls	In the last 6 months, have you fallen in such a way that your body hit the ground? 0 (no)—1 (yes)	No history of falls (0), history of falls (1)	[23]
History of near falls	Are you ever close to falling, but you manage to grab on to something/someone at the last minute so that your body does not hit the ground? 0 (no)—1 (yes)	No history of near falls (0), history of near falls (1)	[6, 24]
Pain	Do you presently suffer from pain? 0 (no)—1 (yes)	No pain (0), pain (1)	[6]
Retropulsion (NRT)	Sudden, unexpected backward pull to the shoulders from behind when standing with feet slightly apart and eyes open; 0 (normal, may take 2 steps to recover)—3 (spontaneous tendency to fall or unable to stand unaided, test not executable)	No retropulsion (0), retropulsion (1–3)	[7]
Tandem gait (TG)	10 heal-to-toe steps along a straight line, with eyes open and without walking aids/support (those using walking aids were asked to try to perform TG without support); 0 (no side steps)—3 (unable to take 4 consecutive steps)	Normal TG (0), abnormal TG (1–3)	[7]

*FOGQsa* freezing of gait questionnaire, *MMSE* mini-mental state exam, *NRT* Nutt Retropulsion Test, *UPDRS* Unified Parkinson’s Disease Rating Scale, *10MWT* 10-m Walk Test

### Follow-up of falls and near falls over 6 months

PwPD were provided with a diary consisting of pre-printed pages for recording the date and time of every fall/near fall event and questions (yes/no) clarifying whether it was a fall or a near fall. The question in relation to a fall was phrased as follows: Did you fall in such a way that your body hit the ground? The corresponding question about a near fall incident was phrased: Were you close to falling, but managed to brace yourself at the last moment (e.g., grabbed on to someone, to an object or the wall)? Falls were defined as “an unexpected event in which the person comes to rest on the ground, floor, or lower level” [23]. Near falls were defined as “a fall initiated but arrested by support from the wall, railing, other person etc.” [24]. The definitions of a fall and a near fall were thoroughly described during the outpatient visit. All participants were telephoned monthly to ensure that registrations had been completed according to instructions. During the last telephone call, they were requested to return the diary in a pre-stamped envelope.

### Statistical analysis

Data were checked regarding underlying assumptions and described accordingly using SPSS version 24 (IBM Corp., Armonk, NY, USA). Normally distributed interval/ratio-level variables were described using means and SDs. In other cases, medians (q1–q3) were used. Categorical variables were described using *n* (%). Wilcoxon signed-rank and McNemar's tests were used for bivariate analysis of differences in sample characteristics between A1 and A2. The alpha level of significance was set at 0.05.

Further analyses were performed through Poisson regression with robust variance. First, simple analyses were performed with each independent variable investigated on A1 and A2 and number of falls and/or near falls investigated at F1 and F2 as a dependent variable. That is, two analyses of short-term predictors were conducted, using A1 and A2 data as independent variables with the number of F1 and F2 falls/near falls events as dependent variables, respectively. Analysis of long-term predictors included A1 data as independent variables with the number of F2 falls/near falls events as the dependent variable. The incidence rate ratio (IRR) values and 95% confidence intervals (CI) were calculated. Variables with a *P* value < 0.20 in the simple analyses were entered in multiple Poisson regression models. The < 0.2 *P* value threshold was chosen to avoid leaving important variables out [26]. Predictors with the highest *P* value were subsequently removed by a backwards stepwise deletion approach until only independent predictors with *P* values < 0.05 remained in the model. Deviance statistic was used to assess the significance of contribution of each independent predictor included in the models. Overall performance of the final models was evaluated with the Omnibus test (where a *P *value < 0.05 suggests adequate goodness-of-fit) and *R*^2^ deviance [27]. For the Poisson regression analyses, all predictor scores were adjusted to be in the same direction (higher score = more problems).

## Results

Fifteen of the 73 PwPD were excluded due to incomplete prospective records of falls and near falls. The baseline mean (SD) age and PD duration of the remaining 58 (32 women) PwPD were 65 (8.8) and 3.2 (3.68), respectively, and their median (q1-q3) UPDRS III score was 10 (6.75–14). Details regarding results from A1 and A2 are provided in Table 2. After 3.5 years, PwPD had more motor symptoms, dyskinesia, and FOG as well as slower comfortable gait speed. In addition, more PwPD reported retrospective falls and near falls, and the number of prospectively reported falls had increased.

**Table 2:** Sample characteristics (*n* = 58)

	Assessment 1	Assessment 2	*P* value
Age (years), mean (SD, min–max)	65 (8.8, 43–80)	69 (8.9, 46–84)	NA
PD duration (years), mean (SD, min–max)	3.2 (3.68, 0.1–17)	6.7 (3.68, 3.6–20,5)	NA
HY stage of disease, median (q1–q3)	2 (2–3)	2 (2–3)	NA
Daily total levodopa equivalent (LDE) dose (mg), median (q1-q3)^c^	200 (300–532)	500 (400–675)	NA
Self-rated motor status at the time of clinical examination			
“on” or “on with dyskinesias”, *n* (%)	56 (97)	48 (83)	NA
“off”, *n* (%)	2 (3)	10 (17)	NA
Motor symptoms (UPDRS III), median (q1–q3)	10 (6.75–14)	13.5 (9–20.25)	< 0.001^a^
Cognition (MMSE), median (q1–q3)	28.5 (27–29)	28 (27–29)	0.129^a^
Abnormal tandem gait (TG), *n* (%)	33 (57)	38 (65.5)	0.302^b^
Dyskinesia (UPDRS IV, item 32), *n* (%)	3 (5)	21 (36)	< 0.001^a^
Comfortable Gait speed < 1.1 m/s (10MWT), *n* (%)	9 (15.5)	24 (41)	< 0.001^b^
Pain, *n* (%)	18 (31)	21(36)	0.481^b^
History of falls, *n* (%)	7 (12)	23 (40)	< 0.001^b^
History of near falls, *n* (%)	17 (29)	37 (64)	< 0.001^b^
Freezing of gait (item 3, FOGQsa), *n* (%)	19 (33)	29 (50)	0.013 ^b^
Retropulsion (NRT), *n* (%)	15 (26)	19 (33)	0.405^b^
Number of falls during 6-month follow-up, mean (SD, min–max)	0.50 (1.53, 0–10)	1.28 (2.05, 0–9)	0.007^a^
Number of near falls during 6-month follow-up, mean (SD, min–max)	2.10 (6.82, 0–45)	1.16 (2.85, 0–16)	0.763^a^
Number of falls and/or near falls during 6-month follow-up, mean (SD, min–max)	2.60 (8.01, 0–50)	2.43 (4.51, 0–25)	0.081^a^

*FOGQsa* Freezing of Gait Questionnaire, *HY* Hoehn & Yahr, *MMSE* Mini-Mental State Exam, *NRT* Nutt Retropulsion Test, *UPDRS* Unified Parkinson’s Disease Rating Scale, *10MWT* 10-m Walk Test

^a^Wilcoxon signed-rank test

^b^McNemar's test

^c^Tomlinson et al. (2010)

### Falls and near falls during 6-month follow-up periods

Twenty-four and twenty-eight PwPD reported at least one fall or near fall during F1 and F2, respectively. The total number of falls was 29 (*n* = 13) during F1 and 74 (*n* = 26) during F2. The mean number of falls (1.28) during F2 was significantly higher (*P* = 0.007) than during F1 (mean, 0.50). The total number of near falls was 122 (*n* = 18) during F1 and 67 (*n* = 19) during F2, and the total number of falls and/or near falls was 151 (*n* = 24) during F1 and 141 (*n* = 28) during F2. Neither the mean number of near falls nor falls and/or near falls differed between the two periods (Table 2).

### Predictors of number of future falls and near falls

#### Short-time predictors

The univariate Poisson regression analyses, based on results from A1 and F1, identified five predictors associated with the number of falls and/or near falls at *P* < 0.2 (Table 3). The multivariate Poisson regression analysis identified a model including only history of falls (IRR, 15.17) as a significant predictor of the number of falls and/or near falls during the following 6 months (Table 3).

**Table 3:** Poisson regression analyses of potential short-term predictors (Assessment 1) of the number of future falls and/or near falls during the following 6 months (*n* = 58)

Independent variables	Participants reporting falls or near falls (*n* = 24)	Participants not reporting falls or near falls (*n* = 34)	IRR (95% CI), *P *value	
			Simple analysis ^a^	Multivariate analysis ^a, b^
Abnormal tandem gait (TG), *n* (%)	18 (75)	15 (44)	1.84 (0.34–9.94), *P* = 0.477	
Cognition (MMSE), median (q1-q3)	28 (27–29.5)	29 (27–29)	0.86 (0.54–1.86), *P* = 0.564	
Comfortable gait speed < 1.1 m/s (10MWT), *n* (%)	7 (30)	2 (6)	0.98 (0.26–3.67), *P* = 0.974	
Dyskinesia (UPDRS item 32), *n* (%)	1 (4)	2 (6)	0.12 (0.21–0.73), *P* = 0.023	
Freezing of gait (item 3, FOGQsa), *n* (%)	13 (52)	6 (18)	4.86 (1.29–18.06), *P* = 0.019	
History of falls, *n* (%)	6 (25)	1 (3)	15.17 (5.08–45.27), *P* < 0.001	15.17 (5.01–45.27), *P* < 0.001
History of near falls, *n* (%)	13 (54)	4 (12)	7.70 (2.31–25.76), *P* = 0.001	
Pain, *n* (%)	9 (38)	9 (26)	1.73 (0.38–7.91), *P* = 0.482	
Retropulsion (NRT), *n* (%)	9 (38)	6 (18)	5.62 (1.54–20.46), *P* = 0.009	

*FOGQsa* Freezing of Gait Questionnaire, *IRR* incidence rate ratio, *MMSE* Mini-Mental State Exam, *NRT* Nutt Retropulsion Test, *UPDRS* Unified Parkinson’s Disease Rating Scale, *10MWT* 10-m Walk Test

^a^For the regression analysis, scores were adjusted to be in the same direction

^b^Multiple Poisson regression analysis (backward stepwise deletion method). Final model goodness-of-fit (Omnibus test), *P* < 0.001; *R*^2^ deviance, 0.459

The univariate Poisson regression analyses, based on results from A2 and F2, identified eight predictors associated with the number of falls and/or near falls at *P* < 0.2 (Table 4). The multivariate Poisson regression analysis identified a model including two significant predictors of the number of falls and/or near falls during the following 6 months: abnormal TG (IRR, 3.77) and lower MMSE scores (IRR, 1.17) (Table 4).

**Table 4:** Poisson regression analyses of potential short-term predictors (Assessment 2) of the number of falls and/or near falls during the following 6 months (*n* = 58)

Independent variables	Participants reporting falls or near falls (*n* = 28)	Participants not reporting falls or near falls (*n* = 30)	IRR (95% CI), *P* value	
			Simple analysis ^a^	Multivariate analysis ^a, b^
Abnormal tandem gait (TG), *n* (%)	22 (79)	16 (53)	4.17 (1.49–11.66), *P* = 0.007	3.77 (1.47–9.67), *P* = 0.006
Cognition (MMSE), median (q1-q3)	27.5 (26.5–28.5)	28 (28–29)	1.19 (1.05–1.34), *P* = 0.006	1.17 (1.03–1.36), *P* = 0.019
Comfortable gait speed < 1.1 m/s (10MWT), *n* (%)	14 (50)	10 (33)	2.33 (0.85–6.44), *P* = 0.101	
Dyskinesia (UPDRS IV item 32), *n* (%)	14 (50)	7 (23)	2.14 (0.84–5.44), *P* = 0.109	
Freezing of gait (item 3, FOGQsa), *n* (%)	20 (71)	9 (30)	2.98 (1.05–8.45), *P* = 0.039	
History of falls, *n* (%)	15 (54)	8 (26)	3.29 (1.38–7.83), *P* = 0.007	
History of near falls, *n* (%)	23 (82)	14 (47)	2.15 (0.75–6.21), *P* = 0.156	
Pain, *n* (%)	10 (38)	11 (37)	1.06 (0.39–2.89), *P* = 0.904	
Retropulsion (NRT), *n* (%)	13 (46)	6 (20)	3.39 (1.46–7.84), *P* = 0.004	

*FOGQsa* Freezing of Gait Questionnaire, *IRR* incidence rate ratio, *MMSE* Mini-Mental State Exam, *NRT* Nutt Retropulsion Test, *UPDRS* Unified Parkinson’s Disease Rating Scale, *10MWT* 10-m Walk Test

^a^For the regression analysis, scores were adjusted to be in the same direction

^b^Multiple Poisson regression analysis (backward stepwise deletion method). Final model goodness-of-fit (Omnibus test), *P* < 0.001; *R*^2^ deviance, 0.079

#### Long-time predictors

The univariate Poisson regression analysis based on A1 and F2 data identified six predictors associated with the number of falls and near falls at *P* < 0.2 (Table 5). The multivariate Poisson regression analysis identified a model including two significant predictors of the number of falls and/or near falls over 6 months 3.5 years later: abnormal TG (IRR, 7.79) and lower MMSE scores (IRR, 1.49) (Table 5).

**Table 5:** Poisson regression analyses of potential long-term predictors (Assessment 1) of the number of falls and/or near falls during 6 months 3.5 years later (*n* = 58)

Independent variables	Participants reporting falls or near falls (*n* = 28)	Participants not reporting falls or near falls (*n* = 30)	IRR (95% CI), *P *value	
			Simple analysis^a^	Multivariate analysis^a,b^
Abnormal tandem gait (TG), *n* (%)	20 (71)	13 (43)	5.53 (2.26–13.49), *P* < 0.001	7.79 (2.90–20.91), *P* < 0.001
Cognition (MMSE), median (q1–q3)	28 (27–29)	29 (27–29)	1.37 (1.12–1.68), *P* = 0.002	1.49 (1.28–1.73), *P* < 0.001
Comfortable gait speed < 1.1 m/s (10MWT), *n* (%)	7 (25)	2 (7)	5.86 (2.67–12.82), *P* < 0.001	
Dyskinesia (UPDRS IV item 32), *n* (%)	2 (7)	1 (3)	3.13 (0.80–12.20), *P* = 0.100	
Freezing of gait (item 3, FOGQsa), *n* (%)	12 (45)	7 (23)	3.12 (1.33–7.28), *P* = 0.009	
History of falls, *n* (%)	6 (21)	1 (3)	1.14 (0.50–2.57), *P* = 0.762	
History of near falls, *n* (%)	12 (43)	5 (17)	2.25 (0.90–5.58), *P* = 0.081	
Pain, *n* (%)	11 (39)	7 (23)	1.60 (0.64–4.00), *P* = 0.316	
Retropulsion (NRT), *n* (%)	8 (29)	7 (23)	1.30 (0.40–4.24), *P* = 0.663	

*FOGQsa* Freezing of Gait Questionnaire, *IRR* incidence rate ratio, *MMSE* Mini-Mental State Exam, *NRT* Nutt Retropulsion Test, *UPDRS* Unified Parkinson’s Disease Rating Scale, *10MWT* 10-m Walk Test

^a^For the regression analysis, scores were adjusted to be in the same direction

^b^Multiple Poisson regression analysis (backward stepwise deletion method). Final model goodness-of-fit (Omnibus test), *P* < 0.001; R^2^ deviance, 0.369

## Discussion

In this prospective cohort study with PwPD, we found history of falls to be a strong short-term predictor of the number of falls and/or near falls in earlier stages of PD, whereas abnormal TG and cognition were identified as both short- and long*-*term predictors in later stages of the disease.

In earlier stages of PD, we found that a person reporting history of falls could be expected, on average, to experience approximately 15 times as many falls and/or near falls during the following 6 months than a person without history of falls. This is in line with prior findings, demonstrating that history of falls is associated with higher frequencies of future falls in PD [1, 14, 15]. Thus, history of falls, the most robust predictor of future falling in PD [28], was confirmed as a strong predictor also of the number of falls/near falls at the earlier course of disease. However, the data generated 3.5 years later yielded a prediction model with abnormal TG as a strongest predictor followed by cognitive decline, whereas history of falls did not contribute to the prediction of the number of falls/near falls over the next 6 months, once TG and cognition were taken into account. This model suggested that the person with abnormal TG could be expected to experience approximately 3.8 times as many falls and/or near falls during following 6 months as a person with normal TG performance. The corresponding value for cognitive decline was 1.2 for a one-unit decrease in MMSE score. The difference in identified short-term predictors in these two models may be related to differences in the frequencies of falls due to disease progression [14]. Indeed, our sample had significantly more motor symptoms and reported more than twice as many falls in the later course of the disease compared to baseline. Based on observations from the baseline assessment and the second follow-up 3.5 years later, these variables were also identified as long-term predictors, suggesting that number of falls and/or near falls is almost 7.8 times higher for a person with abnormal TG and 1.5 times higher for a one-unit decrease in MMSE score. Again, history of falls did not contribute to the long-term prediction once TG and cognition were taken into account.

To the best of our knowledge, the association between TG and frequency of falls/near falls in PD has not been reported before. However, Lindholm et al. reported a prediction model suggesting that abnormal TG is associated with a fourfold higher risk of the occurrence of future falls and/or near falls in early PD [7]. The baseline prevalence of abnormal TG in our sample was high (57%). This result is in line with previous findings [29–31] and suggests that abnormal TG may be a marker of impaired balance in early PD [30].

The critical point of TG is the exact foot placement [32] and ability to keep center of mass (COM) within a very narrow base of support that challenges the mediolateral balance [33, 34]. Normal gait, including ability to walk heel-to-toe, requires that multiple systems, both central and peripheral, are able to function normally and in interaction [35]. Recent results from spatiotemporal analysis of TG suggest that mediolateral balance in PD is impaired compared with healthy controls, and among PwPD with FOG compared to those without FOG [29]. Interestingly, in our study, the prevalence of FOG had increased at 3.5 years, which may have contributed to the associations between TG and the number of falls/near falls. Also, other PD-related complications such as striatal foot deformities, with toe curling, foot inversion, pain, and shortening of muscles [36] may impair TG performance [37]. Furthermore, comorbidities such as vestibular dysfunction, peripheral neuropathies, and age-related musculoskeletal changes are also associated with abnormal TG [35]. All these factors that affect mediolateral balance may also aggravate PD-related postural instability in the lateral direction [38]. Thus, TG may add important information to already existing assessment of postural stability that usually includes shoulder pull in backward direction [21]. This was probably reflected in the strong association between abnormal TG and the number of falls/near falls in our long-term prediction model, and supports the suggestion that abnormal TG may be an indicator of impending disease progression [30].

The second predictor in our models was cognitive decline. Our results suggest that every decrease in the MMSE score results in approximately 1.2–1.5 times higher number of falls/near falls. To our knowledge, this is a novel finding not reported before, although MMSE scores lower than 24 have been associated with an almost sevenfold higher risk of the occurrence of future falls in PD [13].

Some variables that were significant short- and/or long-term predictors of the number of falls/near falls in univariate but not in the multivariate analyses may need a special attention. For example, FOG and comfortable gait speed < 1.1 m/s together with history of falls are included in the three-step falls prediction model [5, 7, 10]. Both FOG and low gait speed may be related to poor TG performance; FOG directly due to PD specific central mechanisms [29] and low gait speed may (in addition to PD-related brady-/hypokinesia) be due to non-PD specific pathologies such as peripheral neuropathies or vestibular dysfunction [39, 40]. These pathologies may aggravate PD-related gait disorders and worsen TG performance [35]. Thus, TG seems to be sensitive to both PD specific and nonspecific aspects of gait and balance disorders in PD, and abnormal TG performance should alert clinicians to consider potential reasons and initiate appropriate therapy.

Taken together, TG and MMSE are both well-known and easily performed clinical tests that predict the number of falls and/or near falls in PD. These tests seem to be stable predictors over time and may also serve as markers of disease progression. They may, therefore, be of special interest to use in clinical settings as predictive screening tests of increased number of falls/near falls. Such information should serve as a starting point for in-depth examination of additional motor and cognitive symptoms such as FOG [29], striatal foot deformities [36, 37], neuropathies [39], dizziness [40], and impaired executive function [11, 41], and may facilitate the rapid implementation of fall prevention strategies [42]. However, further longitudinal studies are needed to explore the external validity of these tests in broader ranges of PD severities and over longer periods of time.

### Limitations

The PwPD in this study had relatively mild PD, and people above the age of 80 years were not included at the start of this study. Our findings may thus not apply to older people or those with more severe PD. However, focusing on PwPD with relatively mild PD has been recommended to work proactively [28, 43].

The number of PwPD that declined participation was high. This means that there is a risk of selection bias and that our sample may not represent the underlying population of PD patients and reduces statistical power and precision in estimates.

Several of the predictors were represented by relatively coarse indicators. Using a coarse indicator, one may not capture those having mild problems. For instance, it has been suggested that the Montreal Cognitive Assessment is preferable to the MMSE when screening for early cognitive impairments in PD [44]. Furthermore, there may well be other predictors of relevance in relation to the aim of this study that were not included in our protocol. However, this is a general limitation with essentially any observational study of this kind, and the included predictors were selected based on clinical experience and what was known from the previous studies at the time.

## Conclusion

In this prospective cohort study with PwPD, we found that abnormal TG and cognitive decline predict the number of falls and/or near falls in the short as well as the long term. History of falls is also a strong short-term predictor but only in earlier stages of the disease. Our observations provide important additions to the evidence base for clinical fall prediction in PD.
